# Progress in the Synthesis and Application of Transparent Conducting Film of AZO (ZnO:Al)

**DOI:** 10.3390/ma16165537

**Published:** 2023-08-09

**Authors:** Dingyi Zhang, Wenhe Yu, Lu Zhang, Xiangyang Hao

**Affiliations:** 1 Engineering Research Center of Ministry of Education for Geological Carbon Storage and Low Carbon Utilization of Resources, Beijing Key Laboratory of Materials Utilization of Nonmetallic Minerals and Solid Wastes, National Laboratory of Mineral Materials, School of Materials Science and Technology, China University of Geosciences (Beijing), Beijing 100083, China; zhangdy97@163.com (D.Z.); wenheyu@email.cugb.edu.cn (W.Y.); 2 School of Environmental Science and Engineering, Qingdao University, Qingdao 266071, China; qduzhanglu@foxmail.com

**Keywords:** AZO thin film, characteristics, preparation process, application

## Abstract

Due to the excellent performance and low cost of the new aluminum-doped zinc oxide (AZO) film, it is expected to replace the mature indium-doped tin oxide (ITO) film. The research status and progress of AZO transparent conductive films are summarized in this review. Moreover, the structure, optoelectronic properties, and conductive mechanism of AZO thin films are also detailed. The thin films’ main preparation processes and the advantages and disadvantages of each process method are mainly discussed, and their application fields are expounded. AZO thin films with multicomponent composite structures are one of the promising development directions in transparent conductive oxide (TCO) thin films. The development of various preparation processes has promoted the production and application of thin films on a broad scale. Finally, some improvement schemes have been proposed to improve the comprehensive performance of the film. The industrialization prospects of the AZO film, as well as its great development potential in the digital world, are discussed.

## 1. Introduction

Transparent conductive oxide (TCO) thin films were discovered in the early 20th century, after which development and research on TCO thin films began. In 1907, Badeker et al. used CdO as a raw material to prepare a transparent conductive coating via thermal evaporation and applied the film to photovoltaic cells, which marked the beginning of their research on TCO thin films. In the 1940s, ultrasonic spray and chemical vapor deposition processes were used to deposit SnO_2_ on glass plates to prepare thin films. Over the past 30 years, researchers have used evaporation and sputtering techniques to deposit In_2_O_3_ and SnO_2_ and fabricate TCO films with improved performance. In 1980, a thin film using ZnO as a raw material accelerated the TCO thin film’s development. With continuous research on the magnetron sputtering process, TCO thin films with low resistivity and high permeability were able to be prepared at low temperatures. In the 1990s [1], some researchers in Japan and the United States began to dope films with two or more oxides to prepare multicomponent compound materials by changing the doping composition and doping amount and manufacture their desired TCO thin film. Indium-doped tin oxide (ITO) thin films were developed and became the mainstream TCO thin films, and magnetron sputtering became the primary preparation process.

TCOs are a class of materials containing In, Sb, Zn, and Cd with optoelectronic properties such as a wide bandgap, high transmittance, and high conductivity [2,3]. TCO films mainly include ITO, fluorine-doped tin oxide (FTO), and aluminum-doped zinc oxide (AZO). Among these, ITO stands out for its excellent optical and electrical properties [4] and wide range of applications in flat-panel display electrodes, solar cells, and organic electroluminescence [5]. However, indium is a relatively expensive scattering element and is less abundant in nature. Plasma from ITO films is unstable when used in solar cells. Therefore, researchers have focused on other films. Currently, AZO thin films are favored by the majority of scientific and technological researchers because of their low cost, non-toxicity, and high stability in H2 plasma [6,7]. Thus far, it has emerged as the most promising thin film material.

Zinc oxide (ZnO) is a hexagonal wurtzite material with a c-axis-preferred orientation. According to the crystallographic model theory, ZnO crystals are formed by the reverse nesting of hexagonal, close-packed oxygen and zinc. ZnO is a wide-bandgap semiconductor with a bandgap of 3.3 eV [8,9]. The intrinsic resistivity of the ZnO film is high. Proper doping not only improves the electrical conductivity of the film but also its stability. ZnO belongs to the II–VI group of compounds, and elements III and VII can replace Zn atoms as doping atoms [10]. Among these doping elements, Al was the most successful and exhibited the best effect. AZO thin films were prepared by doping ZnO with Al using a specific process [11,12]. The microstructures of the prepared thin films were polycrystalline with a wurtzite (hexagonal) structure. In the hexagonal ZnO structure, each Zn atom and the four nearest O atoms formed a tetrahedral structure. Similarly, each O atom and the four nearest atoms formed a tetrahedral structure. Characterization revealed that the films had good photoelectric properties, such as high transmittance and low resistivity. Therefore, thin films have attracted considerable attention for use in AZO.

## 2. Structure and Properties of AZO Thin Films

The structural, electrical, and optical properties of AZO thin films have been studied.

### 2.1. Structure of AZO Thin Films

**Figure 1:**
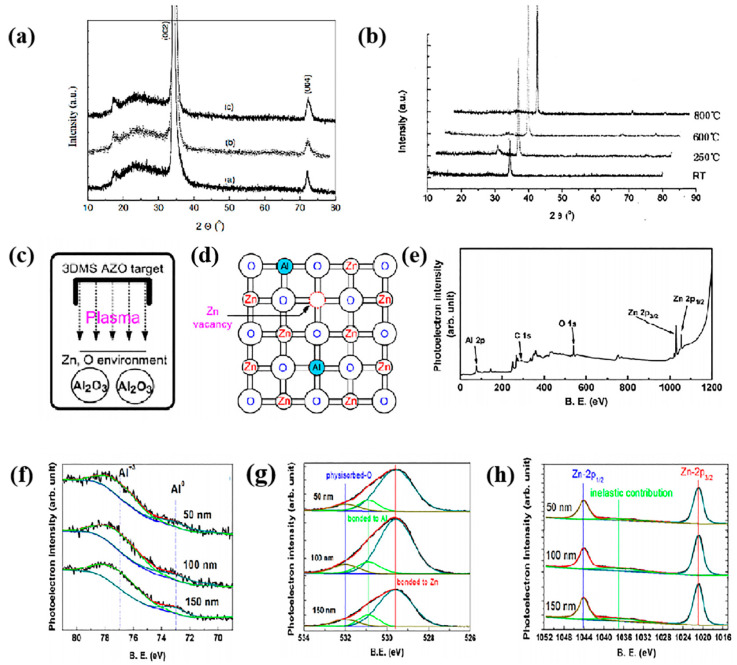
(**a**) X-ray diffraction pattern of AZO films deposited at RT and post-deposition annealed in vacuum at different temperatures [13]: as-deposited, 250 °C, and 450 °C. From WILEY-VCH Verlag GmbH & Co. KGaA, Weinheim, Copyright 2002. (**b**) XRD patterns of ZnO films annealed at different temperatures in air [14]. From Elsevier, Copyright 2005. (**c**) Schematic illustration of 3DMS process plasmas containing O and Zn environment during film growth, (**d**) the possible local lattice structure of the AZO film with the Zn vacancy, (**e**) typical survey scan of the XPS spectrum of the film of thickness 150 nm showing the leading peaks in the AZO films, and (**f**–**h**) core level spectra of Al-2p, O-1 s, and Zn-2p for the selected samples of thickness 50 nm, 100 nm, and 150 nm [15]. From Elsevier, Copyright 2019.

The study of the microstructure of AZO thin films typically begins with their crystal structure and surface morphology. Crystalline structures are typically characterized by XRD. The Al doping only changed the grain shape of the ZnO thin films but not its crystal structure [13]. When Al was doped as a substitute atom for Zn, the thin films exhibited a preferred c-axis orientation along the (002) plane. The polycrystalline structures of films obtained under different conditions differ slightly, leading to changes in the XRD peaks and diffraction angles, which in turn affect their lattice constants. Figure 1 shows the X-ray patterns of the AZO, ZnO, and AZO films.

**Figure 2:**
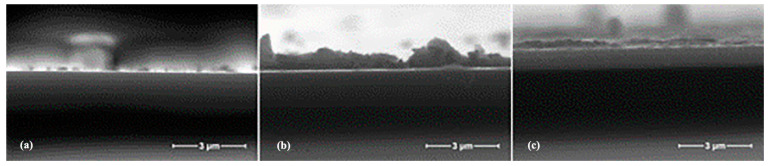
SEM images of the AZO thin films: (**a**) AZO30; (**b**) AZO50; (**c**) AZO400 [16]. From Elsevier, Copyright 2015.

Scanning electron microscopy (SEM) is typically used for surface topography. Nanoparticles were observed on the surface of the ZnO film, and cracks and voids appeared in varying degrees. When the AZO film’s thickness was changed, the holes were significantly reduced, but the roughness values increased. Moreover, the nanoparticles on the surface of the film were uniform and smooth, and their flatness improved. Figure 2 shows the SEM images of the AZO thin films.

### 2.2. Electrical Properties of AZO Thin Films

Although pure ZnO thin films are intrinsic semiconductors, their electrical conductivity is inferior. Adding shallow-level impurities of group III elements Al and Al^3+^ ions into ZnO thin films causes changes in the surface resistivity of the thin films because the conductive electrons of AZO thin films mainly originate from Al atoms occupying the lattice sites of Zn atoms and the absence of oxygen atoms. The number of valence electrons in Al is one more than that in Zn, and weakly bound electrons appeared. When an appropriate concentration of Al atoms is used, many conductive electrons and oxygen vacancies are generated, leading to an increase in the concentration of carriers and a decrease in the resistance rate. An Al:ZnO thin film is an n-type semiconductor, and the Al atoms act as donors and provide electrons to the conduction band. The carrier concentration of the AZO film is 1018–1020 cm^−3^, the electron mobility is 7–100 cm^2^/V·s, and the room temperature resistivity is 10^−2^–10^−4^ Ω·cm. Through some improvement methods, the quality factor of the film can be improved such that the electrical stability is high and the stability can be maintained at room temperature for a long time [17,18].

### 2.3. Optical Properties of AZO Thin Films

AZO is widely regarded as a substitute for ITO in the mass production of photovoltaic equipment owing to its low feedstock cost and photovoltaic properties. AZO has also been successfully applied to organic light-emitting diodes (OLEDs) and LCDs. In the latter case, AZO was used to reduce the thickness of the OLED panels by approximately 10% [19,20].

To analyze the optical properties of AZO thin films, it is necessary to study the visible and infrared light regions. The ZnO film has a forbidden bandwidth of 3.3 eV, which is larger than the energy of visible photons. When irradiated with visible light, it causes intrinsic excitation. Therefore, its transmittance can reach 80–90% in the visible-light region. With the incorporation of Al, the crystallinity of the film changed, and the surface became flat and smooth. It is known that a reduction in grain boundaries and the number of defects reduces the reflection of visible photons and increases the transmittance of the film. Usually, AZO films exhibit a “blue shift” phenomenon (Burstein–Moss effect) because the band structure is closely related to the particle size. With the doping of Al atoms, the crystallinity of the film increased, and the grain size decreased, which caused the absorption band to move in the short-wavelength direction and the emission color to change from red to blue. The researchers found that the “blue shift” phenomenon became more evident with an increase in the doping ratio [21,22,23].

## 3. Preparation of AZO Thin Films

AZO is a recently developed material that has shown potential as a replacement for ITO owing to its good optical and electrical properties. The preparation process parameters determine the crystal orientation, surface flatness, electrical conductivity, optical properties, and gas sensitivity of the AZO films. Various methods have been developed for controlling and improving the properties of AZO films. These methods can be used to obtain high-quality AZO films with high transmittance, high conductivity, good adhesion, and stable performance. AZO films can be prepared using a variety of methods, such as magnetron sputtering [24,25,26], pulsed laser deposition [27,28], sol–gel deposition [29,30], vacuum evaporation [31], and chemical vapor deposition [32,33]. These methods each have their own characteristics. However, they are dedicated to improving film properties, reducing preparation costs, and adapting for commercialization.

### 3.1. Magnetron Sputtering

**Figure 3:**
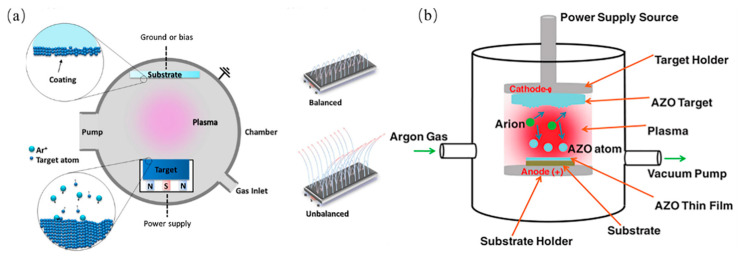
(**a**) Magnetron sputtering scheme and balanced and unbalanced types of magnetron configurations used in MS [35]. From Elsevier, Copyright 2016. (**b**) The schematic diagram for deposition of AZO thin films by RF sputtering technique [37]. From Wiley-VCH GmbH, Copyright 2021.

Magnetron sputtering has been developed since the 1970s and has grown significantly in recent decades to become the procedure of choice for the deposition of a broad range of industrially relevant coatings [34]. Magnetron-sputtered films outperform films deposited by other physical vapor deposition (PVD) processes and may offer the same functionality as much thicker films produced by other surface coating techniques [34]. The setup for magnetron sputtering consists of the following components: electric field (power supply), target plate (cathode), substrate, vacuum chamber, and nonreactive gas (usually Ar) [35]. When a magnetic field is applied near the target area, the gas introduced into the vacuum chamber becomes the target for bombardment by the ionized Ar atoms. The target molecules/atoms were deposited onto the substrate to form a thin coating. Furthermore, radio frequency (RF) magnetron sputtering is considered the most effective deposition technique for thin film growth owing to its ability to modify various deposition parameters, such as the deposition time and RF power, to change the bulk properties of the film [36]. Figure 3 shows a schematic of the deposition of AZO thin films by RF sputtering.

Magnetron sputtering can be classified into direct current (DC), mid-frequency (MF), and RF sputtering. Xia et al. [38] deposited AZO thin films on flexible ultrathin glass substrates using DC magnetron sputtering. Assuming the use of optimum parameters, DC-sputtered AZO thin films on flexible Willow Glass substrates exhibit good properties and will play a significant role in soft and transparent electronic devices. Based on the absorption spectra, we calculated the bandgaps of the AZO films using different sputtering parameters, and the calculated values were in the range of 3.51–3.60 eV. Shi et al. [39] studied the structural, optical, and electrical properties of AZO films prepared by MF magnetron sputtering at room temperature. They found that compressive stress and grain size were dependent on the sputtering power. MF-sputtered AZO films may be suitable for applications in low-cost and flexible optoelectronic devices. Shiravand et al. [40] found that AZO thin films deposited by RF magnetron sputtering had high optical transmittance in the visible region; therefore, these films can be used in TCO materials, making them good candidates for optoelectronic devices.

Yang et al. [41] deposited AZO thin films on quartz substrates using RF magnetron sputtering at room temperature. At an RF power of 300 W, an Ar flow rate of 30 sccm, and a target distance of 7 cm, AZO thin films with a resistivity as low as 4.62 × 10^−4^ Ω·cm, an average light transmittance of 93.7% in the visible range, and a thickness of 250 nm were obtained. Guillén et al. [42] grew transparent and conductive AZO films with thicknesses of 0.3 and 1.1 mm on soda–lime glass substrates by magnetron sputtering at room temperature. At 350 °C, the best properties were obtained after in-vacuo processing of C, where the highest carrier concentration was achieved, with visible-light transmittances of 90–95% and a resistivity of 0.8–0.9 mΩ·cm for AZO layers of different thicknesses.

In general, magnetron sputtering has the advantages of low substrate temperature, high deposition rate, good film adhesion, uniform film thickness, good controllability and stability, simple operation, and low deposition cost. It can also be used for broad-scale production. Magnetron sputtering has been widely used for the industrial coating of architectural glass (low-emissivity coatings), integrated circuits (metal films), flat-panel displays (TCO films), and hard coatings (titanium nitride). Particularly in the field of semiconductor thin-film deposition, people have great enthusiasm for it and will study it further.

### 3.2. Spray Pyrolysis

Compared to magnetron sputtering, spray pyrolysis has many advantages for the preparation of thin films and has attracted much attention in recent years. As the initial technology was simple and inexpensive, techniques for conducting conductive oxide deposition on solar cells and flexible process modifications that allowed for large-scale deposition were developed.

**Figure 4:**
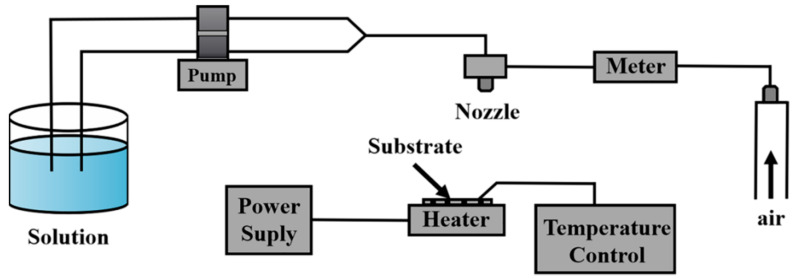
Schematic diagram of the spraying system.

Kaid et al. [43] prepared AZO thin films using spray pyrolysis (SP) of zinc acetate and aluminum nitrate. They found that these films exhibited a polycrystalline texture with a hexagonal structure when deposited on glass substrates at an optimum substrate temperature (TS = 450 °C). Transmission measurements have shown that for visible wavelengths AZO films have an average transmission of more than 90%. Subsequently, the optical parameters were computed. For sputtered films, the dependence of the index of refraction and the extinction coefficient on the wavelength has been reported. The optical gap of AZO is found to be 3.30 and 3.55 eV, respectively, depending on the film thickness. Figure 4 shows a schematic of the spraying system [43].

Pandey et al. [44] successfully produced a TCO, AZO, using ultrasonic spray pyrolysis. They observed several benefits of this method over conventional approaches, including lower equipment costs, excellent thickness uniformity over large areas, and the ability to operate at lower temperatures with reduced vacuum requirements during processing. The resulting resistivity was approximately 10^−3^ Ω∙cm, with a carrier concentration of around 10^20^ cm^−3^, and mobility of roughly 7 cm^2^/V∙s. The optical transmittance of the films exceeded 80%, and the optical bandgap energy increased from 3.25 eV to 3.54 eV.

In spray pyrolysis, the raw liquid materials are ions, polymers, ionic groups, and liquid adhesives. Therefore, the microstructures of complex compounds and solid-solution films can be easily controlled during their formation. However, only Newtonian fluids with low viscosities can be used in spray pyrolysis.

### 3.3. Pulsed Laser Deposition Process

**Figure 5:**
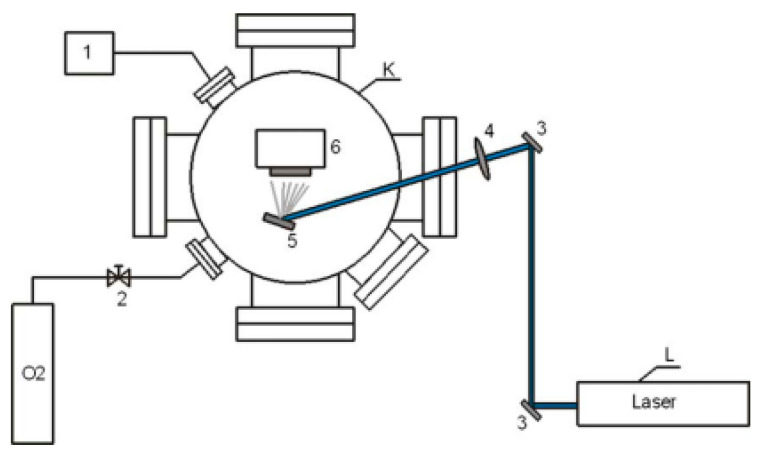
Pulsed laser deposition system for AZO films [46] K—vacuum chamber with turbo-molecular pump, L—laser Nd:YAG, 1—inner pressure control system, 2—O_2_ valve, 3—laser beam track, 4—focusing lens, 5—target, 6—substrate on heated basis. From Elsevier, Copyright 2021.

Pulsed laser deposition (PLD) is an effective method for preparing thin films via vacuum physical deposition and is a very competitive new process. PLD makes use of a high-power pulsed laser beam generated by a pulsed laser on the surface of the target material, causing high temperatures and erosion on the surface of the target material and further generating high-temperature and high-pressure plasma (Tb ≥ 104 K). The domains expand to be emitted and deposited on the substrate to form thin films [45]. Figure 5 shows a schematic of the PLD system for AZO films and its setup.

Anyanwu et al. [47] examined the PLD of AZO thin films at various substrate temperatures. By utilizing a ZnO target doped with 2 wt% aluminum, they achieved a smooth nanostructured morphology for the AZO thin film. The conductivity, transparency, and optical structure of this AZO film exceeded those of AZO and ITO films produced by PLD technology. The superior quality of the deposited film was attributed to the different deposition conditions, particularly the temperature, which had a significant impact on the film characteristics. Consequently, AZO was determined to be a cost-effective and appropriate substitute for ITO. Thus, employing PLD to fabricate highly transparent and conductive nanostructured AZO thin films has potential applications in optoelectronic devices to enable novel functionalities.

Kim et al. [48] successfully generated AZO thin films (3000 Å) on glass substrates with low resistivity and high optical transmittance by utilizing PLD. Specifically, the AZO thin films deposited at a substrate temperature of 200 °C and 5 mTorr oxygen exhibited a resistivity of 3.8 × 10^−4^ Ω·cm and an average transmittance of 91% within the visible-light range. Employing this AZO film as the anode contact in an OLED, they achieved an external quantum efficiency of approximately 0.3% and a current density of 100 A/m^2^. Kek et al. [49] fabricated nanostructures using room-temperature PLD under low-pressure conditions (2.6 Pa) with various background gases such as O_2_, N_2_, He, and Ar at different substrate positions. They observed that the deposition of amorphous AZO films occurred at the top and center positions regardless of the background gas used, whereas at the bottom position, amorphous AZO films were obtained specifically in the presence of O_2_.

Darwis et al. [50] used PLD and matrix-assisted laser evaporation to prepare polymethyl methacrylate (PMMA)-embedded AZO films, which increased the thermoelectric effect by 1.5 to 3 times compared with pure AZO films.

Compared with other processes, the PLD method has the advantages of low deposition temperature, simple operation, independent adjustment of process parameters, precise control of stoichiometry, simultaneous synthesis and deposition, and easy realization of multilayer film growth. The residues of the elementary compounds can be used to deposit high-quality nanofilms and obtain smooth and continuous films on the surface. However, for deposition with more materials, there are target fragments of small molten particles in the film, which can significantly affect the film quality. In addition, the deposition speed is relatively low. When the deposition area is 1000 mm^2^, the deposition thickness is between several hundred nanometers and 1 µm per hour.

### 3.4. Sol–Gel Process

The sol–gel technique is a gentle preparation method that utilizes inorganic substances or metal alkoxides consisting of highly reactive components as precursors. The raw ingredients were thoroughly blended in the liquid phase, followed by hydrolysis and condensation. This process led to the formation of a stable and transparent sol system within the solution. Over time, the sol gradually polymerized between the aged colloidal particles, resulting in the development of a gel with a three-dimensional lattice structure. The liquid components are expelled, leaving behind a gel network. The gel was dried, sintered, and solidified to prepare molecular and nano-substructured materials [51,52,53]. This method is crucial for preparing high-performance particles, fibers, and films.

Islam In et al. [54] fabricated nanocrystalline thin films of undoped ZnO and AZO on a glass substrate using a sol–gel dip-coating technique. They found that the inclusion of the Al dopant reduced the grain size of the films. Khan et al. [55] studied the effect of different Al doping concentrations on the structural, optical, and electrical properties of AZO thin films using the sol–gel method. The 2 mol% AZO film was found to have a maximum bandgap of 3.67 eV, an average transmittance of 84.19%, and a resistivity of 2.05 Ω·cm. Prolonged UV exposure before annealing significantly changed the surface morphology of the film and provided shielding near the UVA (315–378 nm) spectrum, which increased the conductivity of the film by a factor of three and significantly reduced the optical bandgap.

Surajit et al. [56] introduced a sol–gel approach for fabricating AZO transparent conductive films without post-annealing. This method involves the rapid cooling of the glass in the presence of a gas layer, eliminating the additional annealing step. A gas blanket is created by placing AZO-coated glass on a gassing material such as ammonium carbonate, which generates gas through an endothermic reaction. The resulting AZO thin films, with a thickness of 100 nm, exhibit excellent properties, including a low resistivity of 5.4 × 10^−4^ Ω·cm, high transmittance (>90%), and a preferred crystallographic orientation (002). These characteristics are comparable to those of the AZO films prepared using conventional post-annealing techniques in a forming gas atmosphere. The film is quiet. Wang et al. [57] successfully prepared AZO nanoparticles using a novel sol–gel combustion method to investigate the effects of morphology, composition, and sintering activity. The results showed that the crystallinity of the AZO nanoparticles increased with increasing calcination temperature without forming other new phases. The AZO nanoparticles synthesized at pH 4 and 600 °C exhibited the best sintering activity.

Compared to other traditional preparation methods, the sol–gel method has many unique advantages:

Product uniformity. Because this method first dissolves the raw materials in a highly active low-viscosity precursor solution, the reactants can be uniformly mixed at the molecular level within a short period of time when the gel is formed.

High purity. Due to the solution reaction, it is easy to dope and obtain products with higher purity quantitatively and uniformly.

The sol–gel process operates at low temperatures owing to the nanometer range of component diffusion within the sol–gel system, in contrast to the micrometer range observed in solid-phase reactions. Consequently, the desired results can be achieved at reduced temperatures.

Easy preparation of new materials. The material preparation process is easy to control, and new materials that are difficult to obtain using traditional methods can be prepared by selecting the appropriate conditions.

However, the sol–gel method still has disadvantages, such as the high costs of the organic compounds used as raw materials and the adverse effects of organic solvents on the human body. The sol–gel method requires a long time (usually a few days or oven weeks) to process a sample. In addition, gas escapes during the drying process, leaving small gaps and shrinking the surface of the film, making the product easy to crack. Currently, in view of these problems, the following improved process is used: the gel is dried under critical temperature and pressure conditions in the drying medium to maintain the original structure and state of the material against shrinkage and cracking. The precursor was changed to an inorganic metal salt, which effectively reduced the cost of the raw materials.

### 3.5. Vacuum Evaporation

**Figure 6:**
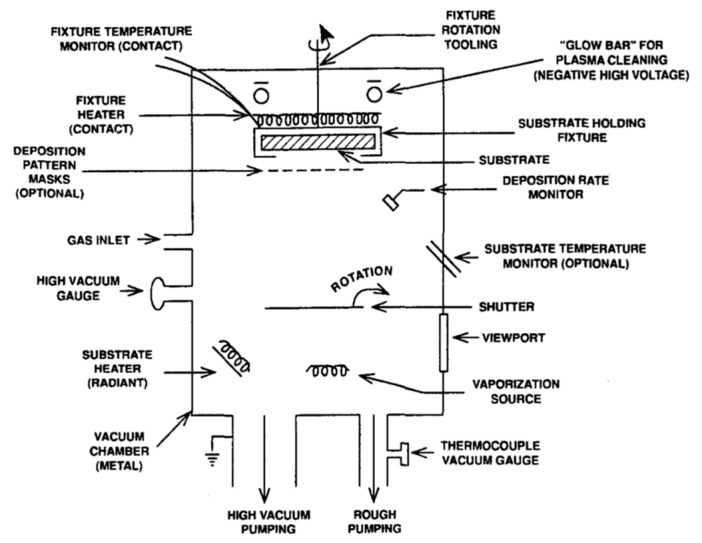
Principal components of a vacuum evaporation system [60]. From Elsevier, Copyright 2002.

Vacuum evaporation refers to the placement of the material under vacuum conditions and the use of a specific heating evaporation method to evaporate or sublime the coating material such that it flies to the surface of the substrate to condense and form a film. Vacuum evaporation is the mainstream process in vacuum plating, and the thermal evaporation rate can be very high compared to other PVD processes [58,59]. Figure 6 shows principal components of a vacuum evaporation system [60].

Vyas et al. [61] introduced the deposition of pure and AZO (1, 3, and 5 at%) thin films on silicon and glass substrates by vacuum evaporation. The results showed that the AZO thin films were composed of more grains than the pure ZnO. The density of the grains increased with increasing Al concentration (from 1 to 5 at%) and the transmittance in the visible region is greater than 90%. Finally, it was found that the optical bandgap initially increased and then decreased with an increase in the atomic concentration of Al and reached a maximum value when the Al concentration was 3 at%. Abdulmunem et al. [62] mixed different weight ratios of aluminum (2, 4, and 6 wt%) and zinc powder (as the target material) in a vacuum quartz tube to deposit thin films on glass substrates via vacuum evaporation. Both pure ZnO and ZnO: Al were found to have a polycrystalline composition, and the preferential orientation of all films was (101). The nanoparticles became more conical with increasing doping ratio and showed decreased optical transmittance and energy gap values, while ZnO and ZnO: Al occurred at the Fermi level at E_f_ = 0.427 eV and E_f_ = 0.667 eV, respectively.

The vacuum evaporation method is a simple film-forming method that can be used to prepare a large-scale film. The required device is relatively simple, the process parameters are few, the thickness of the film can be controlled to prepare a micron or even nanoscale coating quality, and the corrosion resistance is relatively excellent. However, the evaporation process is challenging to control, the repeatability of the experiment is poor, and the degree of vacuum directly affects the film quality. If the vacuum is too low, it will lead to poor adhesion and poor film performance.

### 3.6. Atomic Layer Deposition

The vacuum evaporation method is a simple film-forming method that can be used to prepare a large-scale film. The required device is relatively simple, the process parameters are few, the thickness of the film can be controlled to prepare a micron- or even nanoscale coating quality, and the corrosion resistance is relatively excellent. However, the evaporation process is challenging to control, the repeatability of the experiment is poor, and the degree of vacuum directly affects the film quality. If the vacuum is too low, it will lead to poor adhesion and poor film performance [63].

### 3.7. Chemical Vapor Deposition Process

Chemical vapor deposition technology plays a vital role in many technical fields. When various gases are introduced into the reaction chamber, the chemical reaction of compounds or elemental substances in the chamber occurs on the surface of the substrate, and the generated solid products are deposited on the surface, forming a thin film. This process is a new technology for preparing inorganic materials that has been developed in recent decades.

**Figure 7:**
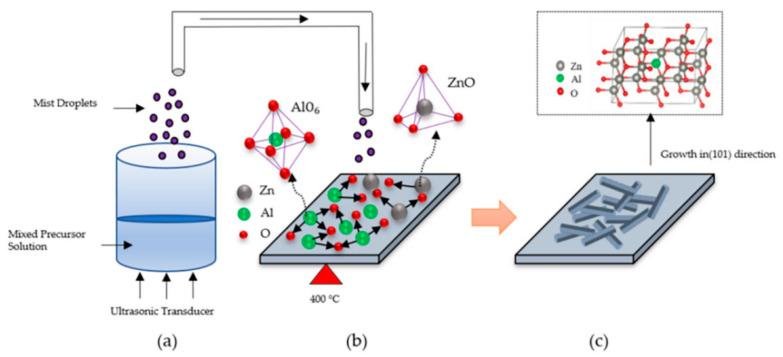
The growth model of AZO films deposited with different Al ratios by the mist CVD method [65]: (**a**) mist droplets generation, (**b**) nucleation, and (**c**) growth process. From MDPI, Copyright 2022.

Saini et al. [64] developed oxide films for thermoelectric orientation using mist chemical vapor deposition (CVD) technology. Two-percent AZO nanoporous films were also fabricated. These nanoporous films enhance phonon scattering and decrease thermal conductivity (κ) while maintaining properties similar to those of dense films fabricated by vacuum techniques. Wai et al. [65] used atomized chemical vapor deposition to deposit AZO films on glass substrates. The variation in the structure of the AZO films was found to be strongly dependent on the Al/Zn ratio. When the deposition temperature was 400 °C, and the Zn/O ratio was greater than the Al/O ratio, the interwoven nanosheet structure was obtained, and the transmittance of all AZO films in the visible region was greater than 80%. The highest photocatalytic efficiency was observed between the wavelengths of 475 and 700 nm. Figure 7 shows the growth model of AZO films deposited with different Al ratios by the mist CVD method [65].

**Figure 8:**
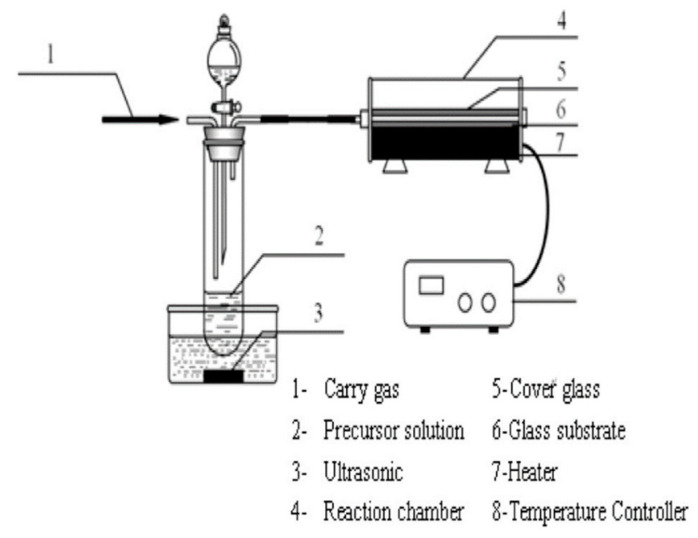
Schematic structure of the AACVD reactor system for ZnO thin film preparation [66]. From Elsevier, Copyright 2016.

Liu et al. [66] used aerosol-assisted chemical vapor deposition (AACVD) to prepare AZO thin films. Figure 8 shows schematic structure of the AACVD reactor system for ZnO thin film preparation [66]. The AACVD technique is an enhanced alternative to conventional CVD, eliminating the need for expensive targets, excessive energy consumption, and high-vacuum conditions [67]. Furthermore, AACVD offers a broader range of high-quality CVD products by utilizing volatile precursors. Additionally, it reduces maintenance expenses because multiple chemical sources can be transported via a single line rather than separate lines. At 450 °C, Potter et al. [68] deposited AZO thin films from zinc acetylacetonate and aluminum chloride by AACVD. The precursor solutions consisted of a mixture of methanol and a secondary solvent, including toluene, tetrahydrofuran, n-hexane, cyclohexane, and ethyl acetate. The results showed that the more polar the solvent, the better the surface growth of the wurtzite crystal structure (002), while a solution with low viscosity could promote grain growth.

CVD technology has excellent application prospects. This technology has a fast deposition speed, easy control of film composition, good adhesion, compactness, and uniformity of the obtained film. Some notable film layers also have good optoelectronic properties, making it easy to achieve mass production. However, the high process temperature of CVD technology can quickly reduce the mechanical properties of the material and weaken the bonding force between the film and substrate, which significantly reduces the product quality. Table 1 shows the advantages and disadvantages of the different methods for the synthesis of AZO films. In summary, the combination of multiple technologies provides guidance for improving efficiency and avoiding shortcomings.

## 4. Applications of AZO Thin Films

### 4.1. Application of AZO Transparent Conductive Films in Solar Cells

At present, transparent solar cell electrodes generally use transparent conductive films with wide bandgaps, such as In_2_O_3_, SnO_2_, and AZO films. In a solar cell, the transparent conductive film acts as a layer to transport carriers from the compartment to the external circuit to achieve the power supply function. AZO thin films have excellent electrical and optical properties, low cost, non-toxicity, and high stability in hydrogen plasma, making them suitable for optoelectronic devices. There is considerable scope for the development of thin-film solar cells [69]. Badgujar et al. [70] fabricated AZO thin films on a 300 mm × 300 mm glass substrate using cold magnetron sputtering. It exhibited a sheet resistance of 8.8 Ω/mm^2^, a visible-light transmittance of 78.5%, and a uniform thickness. The AZO film obtained by optimizing the process conditions was successfully applied as a transparent electrode in solar cells, with a power conversion efficiency of 11.8%.

**Figure 9:**
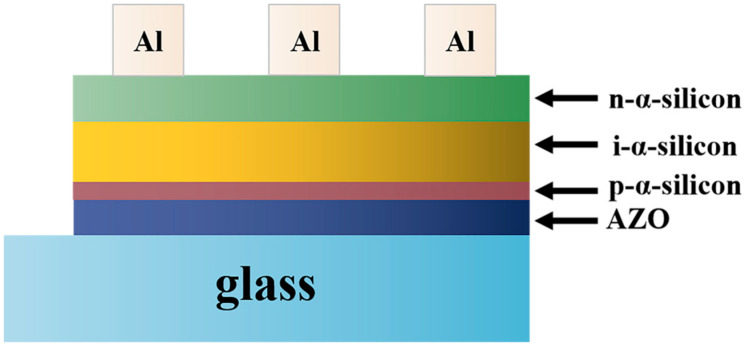
Structure of the superstrate p–i–n α-Si:H thin-film solar cells.

However, there are still some problems with improving solar cell performance by optimizing the performance of AZO thin films. Lin et al. [71] studied AZO films prepared via etching with various etchants. The short-circuit current of the solar cells etched with HNO_3_ was 16.2% higher than that of the naturally grown AZO films, and the conversion efficiency was even higher, reaching 20.2%. In addition to pure AZO thin films, AZO thin films with multilayer composite structures have been widely used to improve the efficiency of solar cells owing to their superior photoelectric abilities [72]. Figure 9 shows structure of the superstrate p–i–n α-Si:H thin-film solar cells.

### 4.2. Application of AZO Transparent Conductive Films in Light-Emitting Diodes (LEDs)

**Figure 10:**
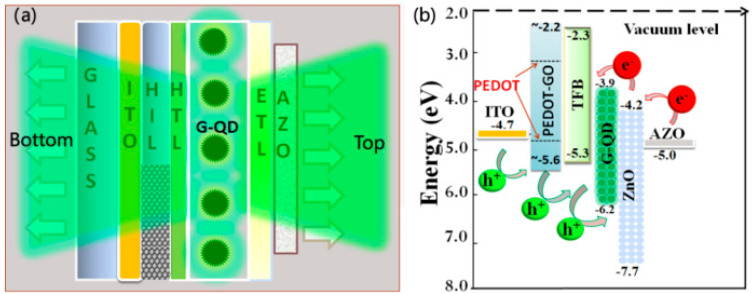
(**a**) A schematic of the dual emissive device structure and (**b**) corresponding energy levels [73]. From MDPI, Copyright 2022.

Quantum-dot light-emitting diodes (QLEDs) have the advantages of a tunable wavelength bandgap in the visible-light range, a narrow half-peak width, and solution preparation. They show great application potential in flat-panel displays and solid-state lighting and have received widescale attention. ITO films are commonly used as anode materials in QLEDs. However, indium and tin are toxic, and raw materials are scarce, resulting in significantly increased production costs. Therefore, researchers have begun focusing on other transparent conductive films. AZO films have gradually emerged as essential transparent conductive films because of their high work function, stable performance, and low resistivity. In addition, applying AZO thin films with a high work function to QLEDs reduces the hole injection barrier, which is beneficial for improving the injection efficiency and balancing the carrier transport in the device. Chen et al. [73] reported a green double-emitting QLED using AZO thin films to tune the band shift between the cathode and the QD emissive layer. The maximum brightness of the fabricated QLED was 9450 cd/m^2^, which corresponds to a power efficiency of 15.7 lm/W, current efficiency of 25.5 cd/A, and a turn-on voltage of 2.3 V. The performance of the AZO-cathode-based QLED was improved 1.3 times. Figure 10 shows a schematic of the dual emissive device structure and corresponding energy levels. Wang et al. [74] used Mg-doped NiOx (Mg-NiO_x_) nanoparticles as the hole transport layer (HTL) and AZO as the electron transport layer (ETL) to improve the performance of ultrastable all-inorganic full-color QLEDs. On the cathode side, Al doping can effectively reduce the conductivity and interfacial charge transfer, resulting in a more balanced charge injection, which improves brightness and efficiency.

### 4.3. Application of AZO Transparent Conductive Film in Bright Windows

**Figure 11:**
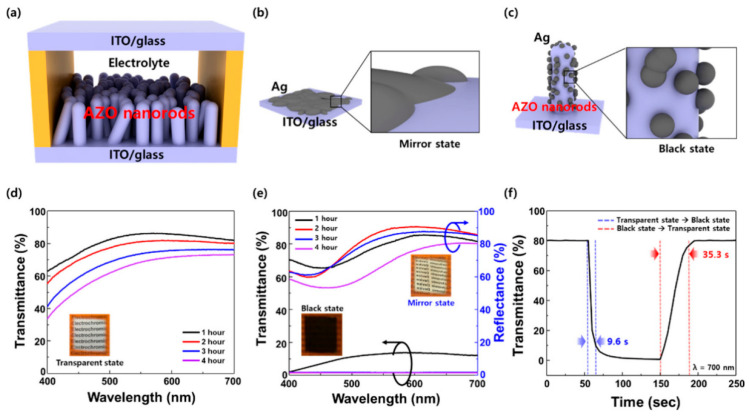
(**a**) Diagram illustrating the configuration of EC windows, comprising a top electrode (ITO/glass), an electrolyte, and a bottom electrode (AZO nanorods/ITO/glass). Representation displaying Ag reduction (**b**) on the ITO/glass and (**c**) on 1D AZO nanorods/ITO/glass. (**d**) The visible spectrum depicts the transmittance (transparent state) and (**e**) reflectance (mirror state) as well as transmittance (black state) of EC windows utilizing AZO nanorods, with growth time as the parameter. The wavelength range for transmittance and reflectance spans from 400 to 700 nm. (**f**) Switching time comparison of EC windows incorporating AZO nanorod_2h_ [75]. From American Chemical Society, Copyright 2022.

In recent years, increasing attention has been paid to environmental protection and energy saving, and “smart windows”, which dynamically regulate solar radiation transmittance, have become a popular research topic. However, current intelligent window materials exhibit high radiation and low transmittance. Combining AZO films with other materials and coating the obtained composite films onto glass can solve this problem. Song et al. [75] presented a highly effective method for enhancing the switching performance of silver-based electrochromic (EC) windows, utilizing one-dimensional (1D) aluminum-doped nanorods (NRs). In Figure 11, they achieved this by subjecting AZO NR electrodes to high-vacuum annealing, resulting in improved surface conductivity and reduced Schottky barrier height (SBH) at the interface. Compared with the switching time of 35.3 s in the EC windows without annealing, the introduction of high-vacuum annealing led to a significant reduction in the switching time of 40%. At visible wavelengths, the EC window exhibited 76.87% transmittance in the transparent state, 1.56% transmittance in the black shape, and 80.19% reflectance in the secular state. It could be used for similar 10,000 cycles without degeneration.

### 4.4. Application of AZO Transparent Conductive Film in Gas Sensor

**Figure 12:**
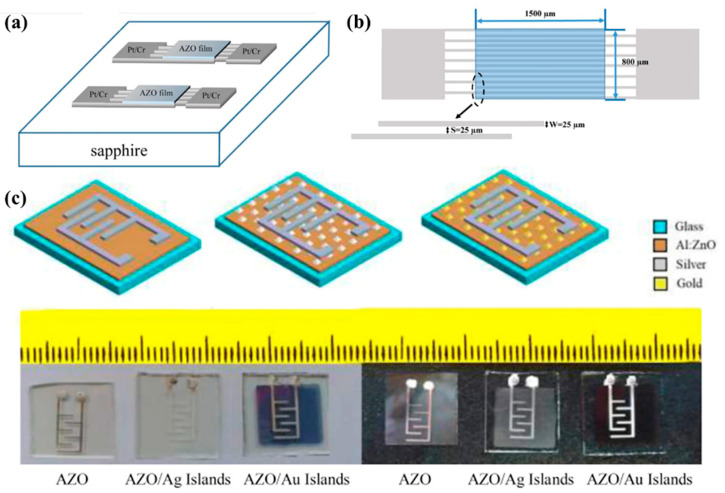
(**a**) Schematic cross-section diagram of the studied devices. (**b**) The geometric designs of the studied device and interdigitated electrodes [12]. From Elsevier, Copyright 2018. (**c**) Ethanol gas sensor configurations: AZO thin film sensor, AZO/Ag active layer sensor, AZO/Au active layer sensor and fabricated samples on glass substrates [78]. From Elsevier, Copyright 2020.

A gas sensor is a device used to detect and control the type and concentration of gas, which plays a critical role in environmental protection and safety. The intrinsic photoconductivity of AZO transparent conductive films changes because of the type of gas adsorbed on the surface; therefore, they can be applied to gas sensors. Sange et al. [76] demonstrated an all-transparent NO_2_ gas sensor based on free-standing hollow AZO nanofibers. Even at low NO_2_ concentrations (0.5 ppm), the sensor responded in a timely manner. Patil et al. [77] deposited nanocrystalline AZO samples on a seeded glass substrate using the reflow method. The gas sensitivity of the AZO sample to 5 ppm NO_2_ gas was 85% at an operating temperature of 175 °C. This work has important implications for applying gas sensor technology at mild and low detection limits. Figure 12 shows schematic cross-section diagram of the studied devices and the geometric designs of the studied device and interdigitated electrodes [12]. In Figure 12, ethanol gas sensor configurations: AZO thin film sensor, AZO/Ag active layer sensor, AZO/Au active layer sensor and fabricated samples on glass substrates [78].

### 4.5. Application of AZO Transparent Conductive Film in Touch Screens

A touch screen is a novel human–computer interaction and inductance liquid crystal display device that can receive input signals. With the continuous development of touch screen technology, AZO transparent conductive films have been applied to touch screens because of their wide bandgap, good electrical conductivity, high transmittance in the visible-light region, and excellent optoelectronic properties. Because their photoelectric properties are similar to those of general transparent conductive thin films and they remain stable in a hydrogen plasma environment, AZO thin films have received extensive attention in the field of touch screens. AZO films are typically compounded with multilayer films and applied to touch screens to improve their performance. For example, in a CNT/Ag/AZO capacitive touch screen, a silver film is deposited on the middle layer so that the high resistivity and rough surface of the CNT can be improved, thereby obtaining a high-performance capacitive touch screen. Lee et al. [79] optimized pulsed DC magnetron-sputtered AZO films using various air/oxygen ratios. The sheet resistance of the optimized AZO was about 600 Ω/square, and the light transmittance was above 83%. The optimized film was applied to a touch panel, showing a light transmittance similar to that of the ITO touch panel and good linearity characteristics.

### 4.6. Other Applications of AZO Films

In addition, the excellent optoelectronic properties of AZO films make them widely used in other fields, such as liquid crystal displays [80,81,82], anti-static films [83], thermal mirrors [84], transparent surface heaters [85,86], and infrared stealth materials [87,88,89], radar shielding protection [90], glucose sensing [91], biosensors [92], antibacterial applications [93], etc. The AZO thin-film material is non-toxic, has a simple preparation process, doping that is easy to achieve, and cheap raw materials that are easy to obtain; therefore, it has great development potential. The field of application will continue to expand with continuous research on AZO thin films.

## 5. Conclusions and Outlook

With the continuous development of flat-panel displays, the large-scale use of solar cells, the constant increase in smart window applications, and the continuous popularization of reflective thermal mirrors, TCO thin films have developed rapidly. AZO thin films have been remarkably developed and applied owing to their excellent optoelectronic properties, non-toxicity, and low cost. In particular, single-layer AZO films have reached a high level of research. However, studies have shown that single-layer AZO films are not as effective as bilayer or multilayer AZO films. Therefore, researchers usually coat a metal film with an AZO film and sometimes deposit an AZO film on it. This double-layer structure, or “sandwich” structure, further improves the AZO film’s conductivity. Thus, the overall performance improves. Due to this, AZO films with cost-effective composite structures are attracting increasing attention.

Current research on the properties of AZO thin films is mainly focused on improving the transmittance of the thin film and reducing its resistance to further reduce the preparation cost; however, there is relatively little research on other properties, such as its ultraviolet or infrared properties and the stability of the internal structure. This also limits the large-scale production and application of AZO thin films because research on these properties is not sufficiently deep. To further improve the comprehensive properties of AZO thin films, future research should focus on the following aspects: (1) the effects of the scattering process of free electrons, dopants, internal defects, and microstructures on the stability and conductivity of the crystals; (2) the effects of varying the annealing temperature and film thickness influences the infrared radiation characteristics of the AZO films throughout the preparation process, determining the best conditions, and preparing low-emissivity films with thermal insulation properties; (3) developing a more competitive production process that is straightforward, less expensive, and pollution-free; and (4) developing low-cost and non-toxic materials, preparing new AZO thin films with composite structures, and expanding the application fields of thin films.

The AZO film has a light-emitting bandgap due to its internal structural characteristics and crystal defects, and the film itself also has light-emitting properties; therefore, the utilization of AZO films in inorganic light-emitting devices represents a potential avenue for further research. Since their invention, AZO films have been extensively studied. It is believed that, with the joint efforts of researchers, AZO thin films will achieve remarkable breakthroughs in performance and expansive application fields.

## Figures and Tables

**Table 1:** The advantages and disadvantages of the different methods for the synthesis of AZO films.

Method	Advantages	Disadvantages
Magnetron sputtering	Low substrate temperature, high deposition rate, good film adhesion, uniform film thickness, good controllability and stability, simple operation, and low deposition cost.	Low in target material utilization rate, plasma instability.
Spray pyrolysis	Easy to control microstructure, high film-forming rate, easy doping, large area film formation.	Only Newtonian fluids.
Pulsed laser deposition	Operating at lower temperatures, control coating density and coating purity, high deposition rate, works on complex shapes.	High equipment cost, slow sedimentary speed.
Sol–gel	Good uniformity of products, high purity, low process temperature, easy to prepare various new materials.	Harmful organic solvents, long production time, cracking easily.
Vacuum evaporation	Excellent corrosion resistance.	Need a decompression device, high viscosity.
Atomic layer deposition	Uniformity of sediment thickness.	The film grows slowly.
Chemical vapor deposition	Fast deposition speed, easy control of film composition, good adhesion, compactness, uniformity of the obtained film.	Reduce the product quality.

## Data Availability

Where no new data were created.
